# Rapid Proteome Changes in Plasma and Cerebrospinal Fluid Following Bacterial Infection in Preterm Newborn Pigs

**DOI:** 10.3389/fimmu.2019.02651

**Published:** 2019-11-15

**Authors:** Tik Muk, Allan Stensballe, Stanislava Pankratova, Duc Ninh Nguyen, Anders Brunse, Per Torp Sangild, Ping-Ping Jiang

**Affiliations:** ^1^Section for Comparative Paediatrics and Nutrition, Department of Veterinary and Animal Sciences, University of Copenhagen, Copenhagen, Denmark; ^2^Department of Health Science and Technology, Aalborg University, Aalborg, Denmark; ^3^Department of Neuroscience, University of Copenhagen, Copenhagen, Denmark; ^4^Department of Neonatology, Rigshospitalet, Copenhagen University Hospital, Copenhagen, Denmark; ^5^Department of Paediatrics, Odense University Hospital, Odense, Denmark; ^6^School of Public Health, Sun Yat-sen University, Guangzhou, China

**Keywords:** Gram-positive infection, proteomics, CSF, plasma, neuroinflammation, sepsis, enteral feeding

## Abstract

**Background:** Neonatal infection and sepsis are common for preterm infants due to their immature immune system. Early diagnosis is important for effective treatment, but few early markers of systemic and neuro-inflammatory responses in neonates are known. We hypothesised that systemic infection with *Staphylococcus epidermidis* (SE), a Gram-positive bacteria, induces acute changes to proteins in the plasma and cerebrospinal fluid (CSF), potentially affecting the immature brain of preterm neonates.

**Methods:** Using preterm pigs as a model for preterm infants, plasma and CSF samples were collected up to 24 h after SE infection and investigated by untargeted mass spectrometry (MS)-based proteomics. Multiple differentially expressed proteins were further studied *in vitro*.

**Results:** The clinical signs of sepsis and neuroinflammation in SE-infected piglets were associated with changes of multiple CSF and plasma proteins. Eight plasma proteins, including APOA4, haptoglobin, MBL1, vWF, LBP, and sCD14, were affected 6 h after infection. Acute phase reactants, including complement components, showed a time-dependent activation pattern after infection. Feeding bovine colostrum reduced the sepsis-related changes in clinical indices and plasma proteins. Neuroinflammation-related neuropeptide Y (NPY), IL-18, and MMP-14 showed distinct changes in the CSF and several brain regions (the prefrontal cortex, PVWM, and hippocampus) 24 h after infection. These changes were verified in TLR2 agonist-challenged primary microglia cells, where exogenous NPY suppressed the inflammatory response.

**Conclusion:** Systemic infection with SE induces inflammation with rapid proteome changes in the plasma and CSF in preterm newborn pigs. The observed early markers of sepsis and neuroinflammation in preterm pigs may serve as novel biomarkers for sepsis in preterm infants.

## Introduction

Preterm infants are at a high risk of bacterial infection in the neonatal period and systemic infections, leading to sepsis, are important for the overall mortality and morbidity of these infants (1, 2). The risk of infection increases with decreasing birth weight and gestational age (GA) (3) and 25–60% of extremely preterm infants (GA <28 weeks) experience at least one infection period in the neonatal period (1). Infections also sensitise preterm infants to non-inflammatory insults, such as hypoxia-ischaemia, and may damage many organs, including the immature brain (1). Infants surviving neonatal sepsis may therefore show increased morbidity related to the respiratory, gastrointestinal, and nervous systems (1, 4–6). Increased susceptibility to infection in preterm infants in the neonatal period can be partly explained by delayed development of the immune system, which may persist into childhood and even adulthood (1, 7). The use of invasive procedures and parenteral nutrition during hospitalisation further predispose preterm infants to systemic infection and inflammation (8).

In neonatal intensive care units, Gram-positive bacteria, such as coagulase-negative staphylococci (CoNS), are often observed as causative pathogens in neonatal infection (3). In surveillance data from the UK, Gram-positive bacteria were causative in 70% of late-onset sepsis (LOS) cases, with 42% due to CoNS (8). *Staphylococcus epidermidis* (SE), a skin commensal bacteria, has been reported to be responsible for up to 50% of LOS cases (9). Unlike Gram-negative bacteria, which triggers the immune response of the host via a lipopolysaccharide (LPS) to activate Toll-like receptor 4 (TLR4), SE triggers the host response mainly via TLR2 (10). In our previous study, bloodstream infection with live *S. epidermidis* caused multiple signs of sepsis in preterm neonatal pigs, such as lethargy, hypotension, respiratory acidosis, internal organ haemorrhage, and neuroinflammation (11). Even without the entry of bacteria into the central nervous system (CNS), Gram-positive infections can cause cerebral inflammation via the activation of TLR2 and are associated with white matter injury and impaired neurodevelopment (10). The systemic administration of a synthetic TLR2 agonist, Pam3CSK4, adversely affects brain development in newborn mice (12), suggesting TLR2 activation has a key role in brain injury associated with Gram-positive infection in neonates.

Prompt diagnosis of infection in preterm infants is difficult as the clinical manifestation is often variable, subtle, and further complicated by non-infectious conditions (13), resulting in delayed or suboptimal treatment. Moreover, antibiotic treatment for treating sepsis alone does not decrease the risk of sepsis-associated cerebral injury (1). Early enteral feeding has been recommended to prevent infections, and antimicrobial proteins and peptides (APPs, e.g., lactoferrin) have been suggested as adjunctive therapies (8). Our previous report showed that early oral feeding of bovine colostrum, rich in bioactive components, can dampen the systemic inflammation caused by Gram-positive infections in preterm pigs (11). In this study, we hypothesised that a Gram-positive infection rapidly alters the inflammatory proteome in the plasma and cerebrospinal fluid (CSF), and enteral feeding mitigates these changes. Sequential plasma collections (6, 12, and 24 h after infection) were used to search for early systemic proteome responses that may provide new targets for more timely sepsis diagnosis and treatment. CSF proteins were profiled to reveal changes pertaining to neuroinflammation in the surrounding CNS. The potential inflammatory effect of selected brain proteins, including NPY, a neuroimmune messenger (14–16) and memory and learning regulator (16, 17), and IL-18, a pro-inflammatory cytokine related to inflammation and blood–CSF barrier integrity (18–20), were further investigated using *in vitro* cell culture systems.

## Methods

### Animal Procedure

**Figure 1:**
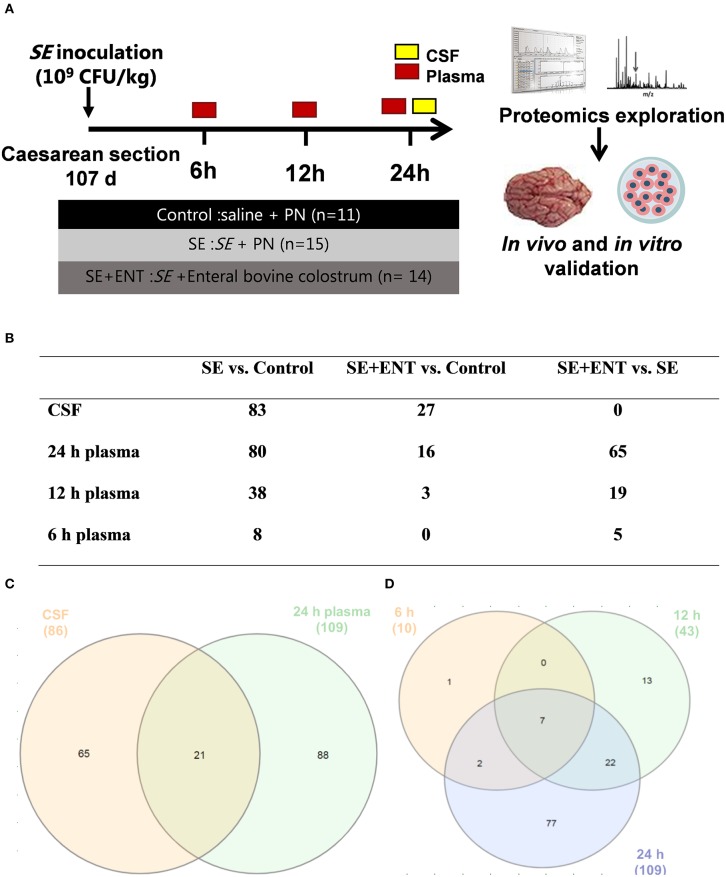
Overview of the animal procedure and the proteomic analysis. **(A)** Animal procedure; **(B)** numbers of samples adopted in the proteomic analysis; **(C)** numbers of proteins with differential abundance in both the CSF and plasma (24 h); **(D)** numbers of plasma proteins with differential abundance at 6, 12, and 24 h post-infection. Numbers in **(C,D)** are numbers of proteins showing difference in any comparison between the treatment groups and are different from those in **(B)** and Table S2 specifying difference between treatment groups.

The delivery, rearing, inoculation, and euthanasia of the premature piglets were carried out as previously described (11) and are schematically presented in Figure 1A. Briefly, preterm piglets (*n* = 40) delivered from two sows (Danish Landrace × Large White × Duroc) via caesarean section at 91% gestation age (107 ± 1 d) were individually incubated with heating (37–38°C) and an oxygen supply. Each piglet was fitted with one vascular catheter (4F, Portex, Kent, UK) into the dorsal aorta via the umbilical cord and an orogastric tube (6F, Portex). Pareteral nutrition and eternal nutrition for each piglet, if applicable, started immediately after the catheterisation. A group of piglets was intra-arterially inoculated with live *Staphylococcus epidermidis* (SE) re-suspended in sterile saline (1.0 × 10^9^ CFU/kg bw; bw, body weight) over 3 min. Fourteen inoculated piglets received enteral feeding of bovine colostrum (10 mL/kg bw/3 h, Biofiber-Damino, Gesten, Denmark) via the orogastric tube and parenteral nutrition (3 mL/kg bw/h) via the umbilical catheter (SE+ENT, *n* = 14). The remaining inoculated pigs received parental nutrition (6 mL/kg bw/h, SE, *n* = 15) only. Piglets that were not inoculated and only received parenteral nutrition (6 mL/kg bw/h) served as controls (Control, *n* = 11). The parenteral nutrition was formulated, as previously described, based on Kabiven (Fresenius-Kabi, Bad Homburg, Germany) and modified to meet the requirement of preterm pigs with added vitamins and minerals (Soluvit, Vitalipid, and Peditrace, Fresenius-Kabi) (11).

At 6, 12, and 24 h post-inoculation, blood was obtained from the umbilical arterial catheter from each piglet for bacteriological, haematological, and blood gas analyses, and EDTA-treated plasma was separated and saved at −80°C for proteomic analysis. Sodium fluorescein (2%, 5 mL/kg bw, Sigma-Aldrich, Brøndby, Denmark) was administered via the umbilical catheter 30 min before the scheduled euthanasia for assessment of blood–CSF barrier permeability. Under anaesthesia, piglets were euthanised with a lethal intracardial injection of sodium barbital (Apotek, Glostrup, Denmark). CSF was directly collected by sub-occipital puncture from each piglet and save at −80°C for proteomic analysis. CSF samples with possible blood contamination, examined by oxyhemoglobin absorbance (λ = 414 nm), were discarded. The number of CSF and plasma samples applied to the proteomic analysis are shown in Figure 1B.

The right hemisphere of the brain was fixed in ice-cold 4% paraformaldehyde for immunohistochemistry (IHC), and different brain parts, including the prefrontal cortex, paraventricular white matter (PVWM), and hippocampus, of the left hemisphere were dissected, snap-frozen, and saved at −80^o^C for later analysis. Animals were treated in compliance with the Animal Experimentation Act of Denmark, which is in accordance with the Council of Europe Convention ETS 123. The study was approved by the Danish National Committee on Animal Experimentation (2014-15-0201-00418).

### Mass Spectrometry (MS)-Based Proteomics

The preparation of protein samples was performed using a filter-aided protocol, as previously described (21). Protein concentration was determined by the absorbance at 280 nm on a NanoDrop Spectrophotometer (Thermo Scientific, Waltham, MA, USA) with bovine serum albumin (BSA) as a standard. CSF or a plasma sample containing 100 μg protein was transferred onto an Amicon Ultra centrifugal filter (10 kDa, 0.5 mL, Millipore, Søborg, Denmark) and mixed with a buffer containing sodium deoxycholate (5%) and triethylammonium bicarbonate (50 mmol/L, pH 8.0). The protein was reduced by TCEP solution [0.5 mol/L, 1:50 (v/v)], alkylated by chloroacetamide [0.5 mol/L, 1:50 (v/v)], and digested by trypsin (Promega, 1 μg/100 μg protein, 37 °C overnight) inside the spin filter with a centrifuge step (14,000 × g for 15 min) in between. Tryptic peptides were recovered by another step of centrifugation and purified by phase extraction using ethyl acetate acidified by trifluoroacetic acid (1%, v/v). Vacuum-dried peptides were suspended in a solution of 2% acetonitrile, 0.1% Formic acid, and 0.1% trifluoroacetic acid and applied onto a Dionex RSLC UPLC System (Thermo Scientific) coupled to a Q-Exactive HF Hybrid Quadrupole-Orbitrap Mass Spectrometer (Thermo Scientific) for analysis. A total of 5 μg of peptide was injected onto a 2 cm reverse-phase C18 material trapping column and separated on a 50 cm analytical column (Acclaim PepMap100, 75 μm ID, 100 Å, Thermo Scientific) with both columns kept at 40°C. The elution gradient was set at a constant flow rate of 300 nL/min and started with a mixture of water (96%) and acetonitrile (4%) containing 0.1% formic acid; it was then increased to 30% acetonitrile over 225 min. Mass spectrometric data were obtained in positive ionisation mode in a data-dependent acquisition fashion with survey spectra and isolation/fragmentation spectra alternating using a top 12 method. Selected peptides were excluded from re-analysis for 30 s. All samples were analyzed in duplicates in a random order.

Protein annotation and quantification based on mass spectra of peptides were carried out using a MaxQuant (version 1.5.2.8) (22) against the UniProt reference database with isoforms (*Sus scrofa*, UP000008227, last modified 2016-08-02) (23). Raw data was searched using an internal re-calibration at 4.5 ppm using two missed cleavages. Cysteine carbamethylation was set as fixed modification and methionine oxidation as variable modification. Allowable missed cleavages were set as 1. The detection of at least two unique peptides per protein and a protein being present in at least 70% of samples in each group were required criteria for protein annotation and quantification. Common contaminants and reverse decoy matches were removed from the annotated protein list. Using the Perseus software (version 1.2.0.17) (24), protein abundance data were normalised and the two-based logarithm transformed to conform to normal distribution and to reduce variability from data acquisition, which was aligned with protein identities and grouping information, such as the treatment and the litter (sow). Aligned data were exported into R (version 3.4.1) (25) and integrated with R Studio (version 3.1.18) (26) for data analysis. The MS proteomic data are available at the ProteomeXchange Consortium (http://www.proteomexchange.org/) with the data set identifier PXD016013.

### Primary Culture of Microglial Cells

The primary culture of microglial cells was prepared as previously described (27) with minor modifications. In brief, hippocampi obtained from preterm piglets from another litter were chopped in ice-cold Krebs-Ringer modified buffer and digested with trypsin for 10 min at 37°C (all from Sigma-Aldrich). Pelleted cells were then washed in the presence of DNase and trypsin inhibitor, re-suspended in Dulbecco's modified Eagle's medium (DMEM) supplemented with 100 μg/mL penicillin, 100 U/mL streptomycin, and 10% heat-inactivated foetal bovine serum (FBS; all from Gibco, Taastrup, Denmark), and kept in cell culture flasks (NUNC, Roskilde, Denmark) to reach confluence. Microglial cells were collected from the supernatant by shaking the culture flask at 250 RPM at 37°C for 4 h. Isolated microglial cells were re-seeded into either 6-well-plates at the density of 25,000 cells/cm^2^ for gene expression analysis or 96-well-plates at the density of 250,000 cells/cm^2^ for ELISA. After overnight rest and growing in serum-free medium for 8–12 h, microglial cells were treated, in duplicates, with either 2.5 μg/mL LTA-SA (from *S. aureus*, InVivoGen, Toulouse, France) or 20 ng/mL Pam3CSK4 (InVivoGen) alone or in the presence of 0.5 μM exogenous NPY peptide (synthetic human NPY, Schafer-N, Copenhagen, Denmark) for 24 h. The level of tumour necrosis factor-α (TNF-α) in the culture was determined in triplicates using porcine DuoSetELISA kits (R&D Systems, Minneapolis, MN, USA).

To estimate the purity of the culture, microglial cells, isolated as described above, were plated into PLL-coated 8-well-Lab-Tek^®^ Permanox chamber slides (NUNC), allowed to grow for 24 h, fixed with 3.7% (v/v) formaldehyde and 1% (v/v) methanol in PBS, and stained with anti-Iba1 antibody (1:2,000, rabbit, ab5076, Abcam, Cambridge, UK) followed by secondary Alexa Fluor 488-conjugated antibody (1:1,000, goat anti-rabbit, Molecular Probes). Cell nuclei were counterstained by DAPI using mounting media (Molecular Probes). Images were recorded using an Axiovert 100 microscope (Zeiss, Jena, Germany) equipped with an AxioCam MRm camera (Zeiss) and ZEN 2012 software (Zeiss). The enriched microglia culture was 90% pure, as determined by the ratio of Iba-1-positive cells to total cells (Figure S1).

### Immunohistochemistry (IHC)

To localise the cerebral neuropeptide Y (NPY) in the brain tissue, brain sagittal sections (5 μm) were probed with primary antibody against NPY (rabbit, 1:3,000, ab30914, Abcam) and a biotin-conjugated secondary antibody (goat-anti-rabbit, 1:500, Vector Labs, Peterborough, UK) after standard deparaffinisation, rehydration, antigen retrieval, and blocking procedure. Staining was visualised with 0.04% 3,3′-diaminobenzidine (DAB, Sigma-Aldrich) using an ABC Peroxidase Staining Kit (Thermo Scientific). For the quantification of NPY expression in brain, whole sections were scanned at a 10 × objective magnification on a Zeiss Axio Scan Z1 (Zeiss). The region of interest was selected in similar anatomical regions across samples, and the number of NPY-positive cells was counted in the defined frames. Representative images were acquired with a BX-51 microscope (Olympus, Ballerup, Denmark) using Integrator System software (Visiopharm).

### ELISA

The concentration of IL-18 in CSF and plasma samples was measured with a porcine IL-18 ELISA kit (RayBiotech, Norcross, Georgia, USA). The frozen tissue of two brain regions, the prefrontal cortex and PVWM, was homogenised and lysed for protein extraction. Total protein concentration was determined by a Pierce™ BCA kit (Thermo Scientific, Waltham, Massachusetts, U.S) for total protein adjustment later. The concentration of NPY in two brain regions was measured with a porcine Neuropeptide Y (NPY) ELISA kit (Phoenix Pharmaceuticals, Burlingame, CA, USA), and the final concentration was adjusted to total protein concentration.

### Gene Expression by Real-Time qPCR

Transcription of selected genes in the prefrontal cortex, PVWM, and hippocampus was determined by real-time qPCR, using predesigned primers (sequences listed in Table S5). Briefly, total RNA in tissue homogenate was isolated with RNeasy Lipid Tissue Mini Kit (Qiagen, Copenhagen, Denmark). RT-qPCR was performed using a QuantiTect SYBR Green PCR Kit (Qiagen) on a LightCycler 480 (Roche, Hvidovre, Denmark). The relative level of target genes was normalised to the housekeeping gene *HPRT1* (28).

### Data Analysis and Statistics

Before analysis, the mean protein abundance of three technical replicates of each sample in the proteomic analysis were combined into one as previously described (29). An univariate analysis was applied to proteomics data in each sample type (CSF or plasma) at each sampling time-point in R. Briefly, a linear mixed-effect model was fitted to each protein with treatment as the fixed-effect factor and litter as a random-effect factor using the lme4 package (30). A Tukey *post-hoc* test (package multcomp) on this model was adopted to compare the effect between three treatment groups, i.e., Control, SE, and SE+ENT, in a pairwise fashion (31). To control the type I error, *p*-values were further adjusted by false discovery rate (FDR, α = 0.2) into q values within each comparison of each sample type (plasma or CSF) at each time-point using the multtest package (32). Proteins with a *q* ≤ 0.10 in any comparisons were chosen for functional assignment, which is generally accepted for this kind of explorative proteomic analysis. Dunn's Kruskal-Wallis Multiple Comparisons test of various clinical data and Pearson's correlation test were conducted in R. Results from RT-qPCR and ELISA were analyzed using a Student's *t*-test and a two-tailed *p* < 0.05 was considered as statistically significant. Data were presented as mean ± SEM unless otherwise stated.

## Results

### Clinical Data

As shown in our previous report, preterm piglets inoculated with live SE developed sepsis and blood–CSF barrier disruption, and enteral feeding ameliorated these damages (11). The clinical variables of the piglets included for proteomic analysis, namely the aSOFA score, leucocyte count in the CSF and blood, platelet count in blood, and CSF–blood fluorescein ratio, are presented in Table S1. Similar to our previous report, the SOFA score adapted to preterm piglets (aSOFA), CSF leukocyte count, blood levels of C-reactive protein (CRP), and fibrinogen at 24 h were elevated after SE infection (all *p* < 0.05), indicating sepsis and elevated systemic and intracranial immune response in the SE piglets. Blood–CSF barrier permeability was higher in SE piglets, as shown by the higher CSF-serum fluorescein ratio (*p* < 0.05) relative to the controls. The blood leukocyte number decreased after birth (*p* < 0.05) in Control, while SE infection maintained the leukocyte count over time (*p* = 0.91), although generally lower than in Controls. The blood leukocyte number in SE + ENT piglets tended to decrease over time (*p* = 0.08), similar to Control, but at a lower level. The platelet number increased over time in all three treatment groups, while the value in SE piglets was lower than that in Control and SE+ENT pigs (*p* < 0.05 for all comparisons between treatment groups, except SE vs. Control at 24 h). The values in SE+ENT pigs were similar to those in Control.

### CSF and Plasma Proteomics

**Figure 2:**
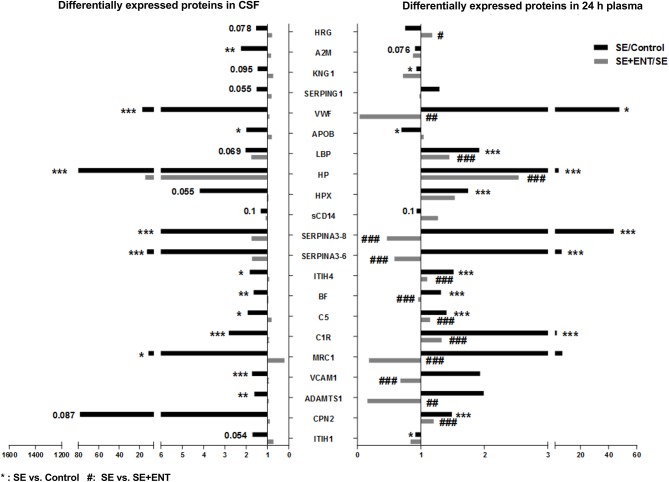
Twenty-one differentially expressed proteins present in the CSF and plasma 24 h post-infection. A majority of these proteins, 15 of 21, present in both the CSF and 24 h plasma were associated with immune response, most of which changed in the same direction. Fold changes of SE/Control and SE+ENT/SE of specific proteins are displayed in bars. ^*^SE vs. Control; ^#^SE vs. SE+ENT; ^*^ or ^#^, *q*, or *p* < 0.05; ^**^ or ^##^, *q*, or *p* < 0.01; ^***^ or ^###^, *q*, or *p* < 0.01.

No significant difference in the protein concentration of CSF samples was observed among the three groups (*P* = 0.17) as tested by a linear model including factors such as treatment, sex, and litter. In CSF and plasma samples, 1,960 and 735 proteins were annotated (as shown in Data Sheets S1 and S2), respectively, and 86 CSF proteins and 114 plasma proteins that met the selection criterion (*q* ≤ 0.1 in at least one treatment comparison) were selected for functional assignment (Figure 1C). The numbers of differential proteins in any comparison in the CSF and plasma at the different time-points are listed in Figures 1B–D and Table S2. Among the selected CSF proteins, 83 showed differential expression between SE vs. Control, while 27 showed differential expression for SE+ENT vs. Control and 1 for SE + ENT vs. SE. In plasma, the number of differential proteins (SE vs. Controls) increased over time, 10, 43, and 109 at 6, 12, and 24 h, respectively (Figure 1D). Twenty-one proteins with differential abundance appeared in both the CSF and 24 h plasma, and proteins, such as serpin G1, von Willebrand factor (vWF), LPS-binding protein (LBP), haptoglobin, hemopexin (HPX), MRC1, and VCAM1, changed similarly in the CSF and 24 h plasma (Figure 2). Seven plasma proteins, including haptoglobin, vWF, serpin a3-8, serpin a3-6, sCD14, LBP, and apolipoprotein A-IV, maintained their differential abundance pattern across three time-points, although at different significance levels (Figure 1D).

According to their major physiological functions, listed in the Uniprot database and publications, differential proteins were categorised into groups including “neurodevelopment (development),” “immune response,” “protein processing,” “metabolism,” and “others.” Observed acute phase reactants were categorised as a separate group. The protein groups may show overlap and are not mutually exclusive. Information on proteins with differential abundance, including UniProt ID, gene name, protein name, and abundance in each group, is shown in Tables S3, S4.

### Immune Response Proteins

In the CSF, 38 differential proteins were related to immune response (45% of all regulated proteins, Table S3), whereas 48 (44%) immune-related proteins were detected in plasma at 24 h, 24 (56%) at 12 h, and 8 (80%) at 6 h (Table S4). All differential immune response proteins, including acute phase reactants, in the CSF were affected by the SE infection (SE vs. Control). None of these proteins, except IL-18 BP, were affected by enteral feeding (SE+ENT vs. SE). Fifteen of 21 proteins present in both the CSF and 24 h plasma were associated with immune response, including A2M, BF, C1R, C5, CPN2, HP, HPX, ITIH1, ITIH4, KNG1, LBP, Serpin A3-8, MRC1, SAA2, and APOB (Figure 2 and Tables S3, S4). Among them, A2M, ITIH1, KNG1, and APOB were elevated in the CSF of SE pigs relative to the Control, but decreased in 24 h plasma. The remaining proteins showed elevated abundance in both types of samples after SE infection (Figure 2). At 24 h, 45 of the 62 differentially regulated proteins in plasma by SE were also affected by the enteral feeding (SE+ENT vs. SE, *q* < 0.1), 16 proteins were affected at 12 h, but only 5 proteins at 6 h.

### Neuropeptide Y (NPY)

**Figure 3:**
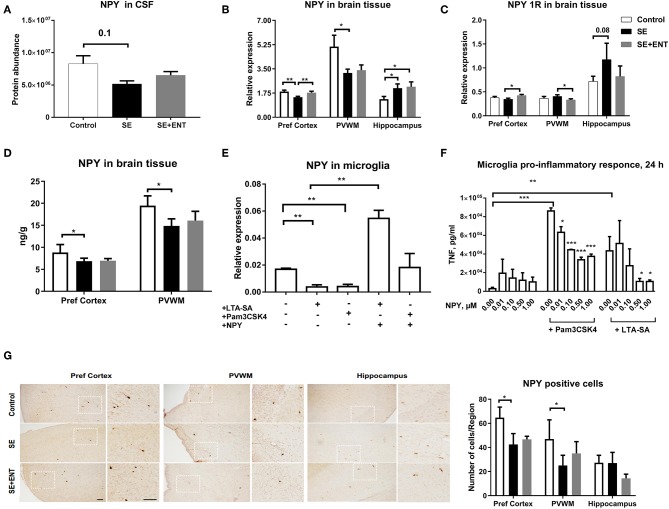
Proteomics, brain transcription, and *in vitro* analyses of NPY. **(A)** Protein level of NPY in CSF decreased post-infection; **(B)** transcription of NPY decreased by SE infection in two brain regions, the prefrontal cortex and PVWM, but increased in the hippocampus; **(C)** transcription of NPY 1R increased by colostrum feeding in prefrontal cortex and PVWM; **(D)** protein level of NPY in significantly down-regulated by SE infection; **(E)** transcription of NPY in cultured primary microglia cells challenged by TLR2 agonists alone and in combination with NPY (0.5 μM); **(F)** secreted TNFα level in primary microglia culture co-treated with LTA-SA, Pam3CSK4, and NPY for 24 h. The TNFα level was decreased by exogenous NPY; **(G)** NPY-positive cells in prefrontal cortex, PVWM, and hippocampus. All data are presented as mean ± SEM. White squares are magnified inserts. Scale bar, 100 μm. ^*^, *q*, or *p* < 0.05; ^**^, *q*, or *p* < 0.01; ^***^, *q*, or *p* < 0.001.

Neuropeptide Y (NPY) was only detected in the CSF samples and not in plasma samples, and it was detected at a lower level in the SE pigs relative to the Control (Figure 3A). In the brain tissue, *NPY* transcription is lower in the prefrontal cortex and PVWM in SE piglets relative to the Control, while enteral feeding increased the transcription of *NPY* and one of its key receptors, *NPY 1R*, in the prefrontal cortex (Figures 3B,C). Unlike that in the prefrontal cortex, transcription of *NPY* (*p* < 0.05) and *NPY 1R* (*p* = 0.08) in the hippocampus increased after SE infection, but no effect of enteral feeding was observed. The protein level of NPY in two brain regions, the prefrontal cortex and PVWM, was significantly lower in SE piglets, which was consistent with the transcription and proteomic data (Figure 3D). Immunohistochemical staining for NPY in selected brains confirmed its presence within the same brain regions (Figure 3G).

In a previous study, the immuno-positive brain area against Iba-1, a marker for microglial cells, significantly increased in the SE group compared with the controls, but it was not significantly affected by the colostrum feeding (11). Therefore, to investigate the direct response of microglial cells to acute SE stimulation, a primary porcine cell culture was employed. In this culture, stimulation for 24 h with TLR2 agonists (LTA-SA, Pam3CSK4) decreased *NPY* transcription (*p* < 0.01, Figure 3E) but increased the transcription of *NPY 1R* (only by Pam3CSK, *p* < 0.05, Figure S1B). Treatment with exogenous *NPY* upregulated the transcription of *NPY* (vs. LTA-SA alone, *p* < 0.01, Figure 3E) and *NPY 1R* (vs. Pam3CSK4 alone, *p* < 0.05, Figure S1B). Further, exogenous NPY (0.01, 0.10, 0.50, and 1.00 μmol/L) dose-dependently suppressed the production of TNF-α triggered by TLR2 agonists (Figure 3F).

### IL-18, IL-18 BP, and MMP-14

**Figure 4:**
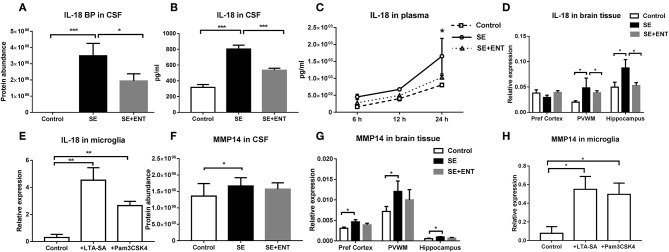
IL-18 BP, IL-18, and MMP-14 in CSF, plasma, brain tissue, and cultured microglia. **(A)** IL-18 BP level in the CSF detected only in SE and SE+ENT piglets; **(B,C)** IL-18 levels in the CSF and plasma, both significantly upregulated by SE infection; **(D)** transcription of IL-18, significantly increased in the SE group in three brain regions and decreased by enteral feeding of colostrum in the PVWM and hippocampus; **(E)** transcription level of IL-18 in primary microglia, significantly increased by the treatment of TLR2 agonists; **(F)** protein level of MMP-14 in the CSF, increased in SE pigs; **(G)** transcription level of MMP-14 in three brain regions, increased by SE infection; **(H)** transcription level of MMP-14 in primary microglia, increased by the treatment of TLR2 agonists. Data are presented as mean ± SEM. ^*^, *q*, or *p* < 0.05; ^**^, *q*, or *p* < 0.01; ^***^, *q*, or *p* < 0.001.

In the CSF, IL-18 binding protein (IL-18 BP) was only detected in the SE and SE + ENT groups, with the highest level being in SE pigs (*q* < 0.05, Figure 4A). Levels of IL-18 in the CSF and plasma (24 h) of SE piglets, determined by ELISA, were elevated when compared with the Control (both *p* < 0.05, Figures 4B,C), but enteral feeding decreased IL-18 levels (both *p* < 0.05, Figures 4B,C). In the hippocampus and PVWM, *IL-18* transcriptions were higher in the SE than in the Control and SE+ENT pigs (all *p* < 0.05, Figure 4D). In the primary microglial culture, *IL-18* expression was upregulated by TLR2 agonists (*p* < 0.01, Figure 4E).

The CSF level of MMP-14 was higher in SE piglets than that in Control (q <0.05), while no significant difference was observed between SE+ENT and SE piglets (Figure 4F). Similarly, *MMP-14* transcription increased relative to the Control in the brain of SE piglets as well as in TLR2 agonist-challenged primary microglial cells (all *p* < 0.05, Figures 4G,H).

### Acute Phase Reactants

Multiple acute phase reactants were detected in the CSF and plasma after SE infection, including those related to opsonisation (CRP, SAP, C1q), SAA, coagulation factors (fibrinogens, factor VIII and vWF), α-2-macroglobulin, microbial iron uptake-inhibiting haptoglobin, and complement components and various serpins including α-1-antitrypsin (Table S4). These “positive” acute phase reactants increased post-SE infection, while multiple ‘negative’ acute phase reactants decreased, including albumin, transferrin, transthyretin, and retinol-binding protein 4 (Table S4).

**Figure 5:**
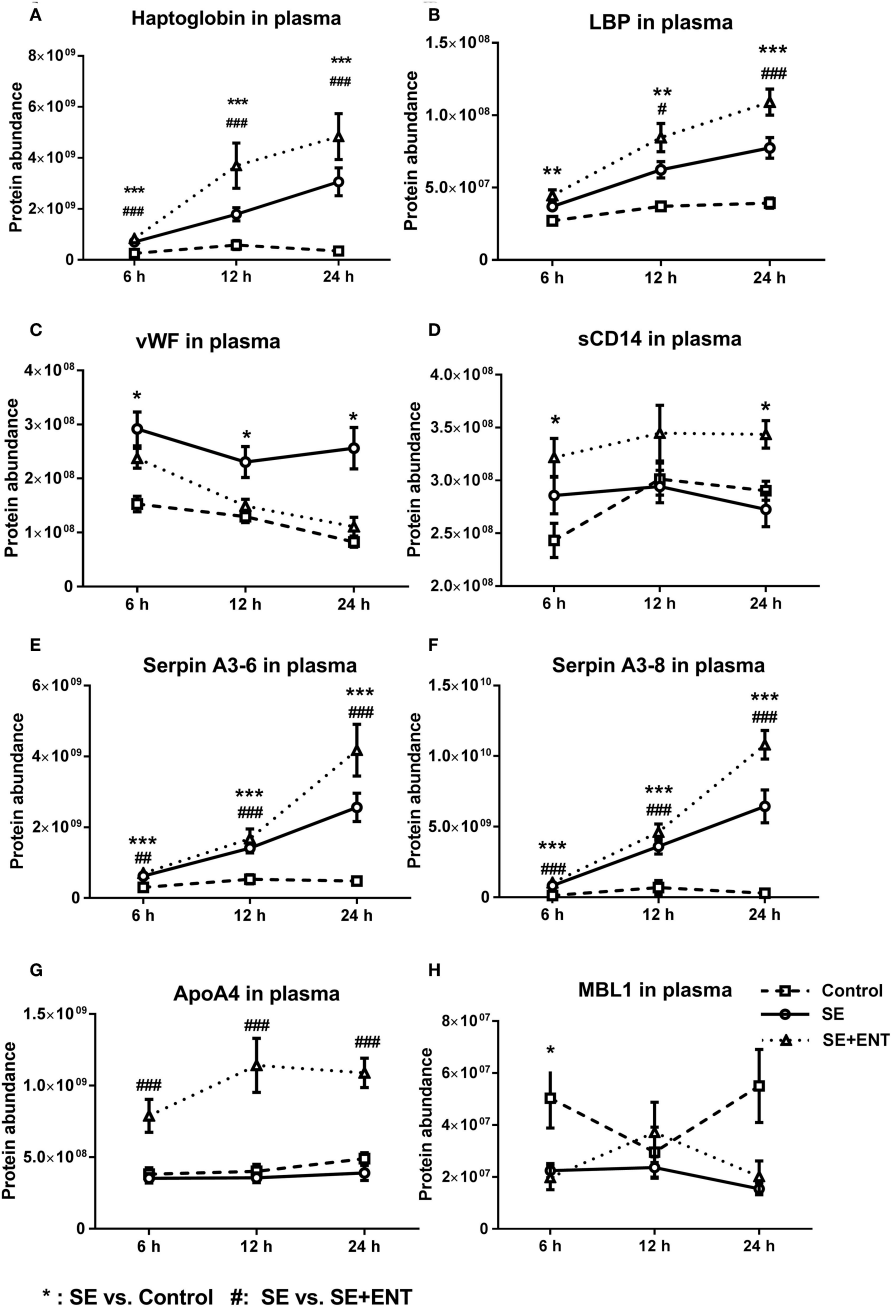
Abundance change of plasma proteins over time. **(A)** haptoglobin, **(B)** LBP, **(C)** vWF, **(D)** sCD14, **(E)** Serpin A3-6, **(F)** Serpin A3-8, **(G)** ApoA4, and **(H)** MBL1. Immune response proteins increased in abundance as early as 6 h post-SE infection, and most of those proteins maintained this change until 24 h. Data are presented as mean ± SEM. ^*^SE vs. Control; ^#^SE+ENT vs. SE; ^*^ or ^#^, *q*, or *p* < 0.05; ^**^ or ^##^, *q*, or *p* < 0.01, ^***^ or ^###^, *q*, or *p* < 0.01.

**Figure 6:**
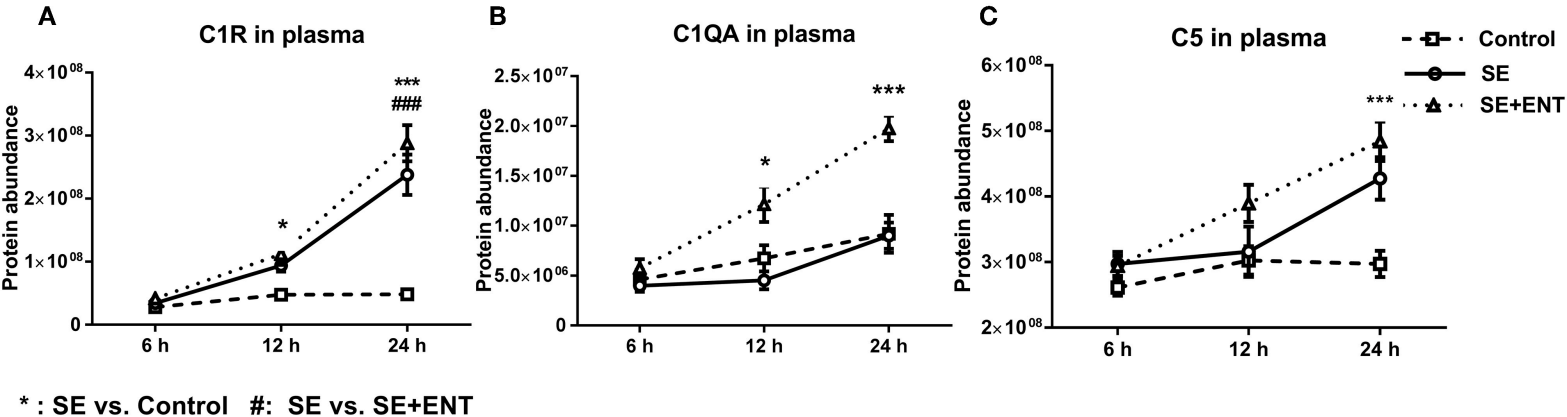
Plasma level of complement components. **(A)** C1R, **(B)** C1QA, **(C)** C5, C1QA, C1R, and C5 showed change 12 h after SE infection, and C2, C4BPA, C5, and C8B showed changes 24 h after infection. Data are presented as mean ± SEM. ^*^SE compared to Control; ^#^SE+ENT compared to SE; ^*^or ^#^*q* < 0.05; ^**^ or ^##^*q* < 0.01, ^***^ or ^###^*q* < 0.01.

Multiple immune response proteins, including Serpin A3-6, Serpin A3-8, MBL1, vWF, haptoglobin, LBP, and sCD14, increased in abundance as early as 6 h post-SE infection. Most proteins, including LBP, vWF, Serpin A3-6, and Serpin A3-8, maintained this change until 24 h (all p < 0.05, Figure 5). In 24 h plasma, C1QA, C1R, C2, C5, C6, C8B, and C4BPA increased in SE piglets relative to the Controls. Increases in C1R, C1QA, and C5 levels were observed in 12 h plasma as well (all *p* < 0.05, Figure 6). However, no C3 and C4 were observed with differential abundance in plasma within 24 h. ApoA4 increased significantly in the SE+ENT group from 6 h to 24 h when compared with the Control and SE groups (all *p* < 0.001, Figure 5).

## Discussion

In this study of systemic infection with SE-induced sepsis and neuroinflammation, preterm pigs were used as a model for preterm infants. Within 24 h of SE infection, a large number of proteins related to the immune response were affected in the CSF and plasma, and a number of proteins (genes) had their expression affected in various brain regions. Enteral feeding with bovine colostrum modified the proteome response in plasma within 24 h, while less CSF proteins were affected within this time frame. Collectively, these results suggest that plasma and CSF proteins may be a rich source of early biomarkers for sepsis and neuroinflammation in preterm neonates. Furthermore, they show that early enteral feeding may be important in dampening the systemic and brain inflammatory response to infection.

Besides activating leukocytes, the systemic immune system may enlist proteins supporting immune response to enter the CNS. In the current study, 15 immune response-related proteins changed in abundance in both the CSF and plasma following SE infection, and most of them changed in the same direction. The majority of these proteins were related to immune defence, for example, KNG1 was involved in blood coagulation and platelet degranulation. Other proteins were involved in acute phase response, such as A2M, properdin, C1R, C5, CPN2, haptoglobin (inhibition of microbial iron uptake), serpins (inter-α-trypsin inhibitors), and SAA (immune cell recruitment). LBP and sCD14 (presepsin), involved in TLR4 activation, were also stimulated by SE infection (Table S4), confirming results from newborn infants (33); a Gram-negative infection, however, may induce even a higher level of sCD14 than a Gram-positive infection (34). The SE-elevated proteins found in the CSF may partly originate from the systemic circulation and travelled through a more permeable blood–CSF barrier in these preterm pigs. Of many proteins, enteral feeding of bovine colostrum only affected the plasma level (Tables S3, S4), suggesting that enteral feeding, at least with bovine colostrum, mainly affected the systemic inflammation and had a limited effect inside the CNS, at least in this short term.

The brain inflammatory response was demonstrated by the SE-induced change in abundance of 38 CSF proteins that were related to neuroinflammation and 23 proteins related to neurodevelopment, including glial cell differentiation, astrocyte migration, and synaptic adhesion (Table S3). Among these proteins, neuropeptide Y (NPY), a neuropeptide in the CNS with pleiotropic roles in neurogenesis, neuroprotection, and neuroinflammation (35), showed decreased abundance in the CSF after SE infection. NPY mediates the interaction between the nervous and immune systems (35) and may be involved in the activation of resident immune cells; microglia and its upregulation may counteract inflammatory processes (36). In the current study, differential expression of *NPY* in three brain regions of SE-infected pigs (the prefrontal cortex, PVWM, and hippocampus) indicates that brain-derived NPY, at least in part, contributes to the altered NPY level in SE-infected CSF. On the other hand, circulating NPY may also have contributed to the NPY levels in the CSF, especially in the light of the elevated blood–CSF permeability post-SE infection. Unlike that in the prefrontal cortex and PVWM, the hippocampal expression of *NPY* was elevated in the SE group. Besides, elevated transcription of *NPY 1R*, one key receptor of *NPY*, was only found in the hippocampus of SE piglets, supporting other studies (37). Our results suggest that SE infection triggers highly distinct responses in different brain regions of preterm neonates.

Microglial cells, the resident macrophages in the brain, provide an immune defence that involves TLRs and are a key source of NPY production (35). Primary porcine microglial cells decrease their expression of *NPY* when treated with TLR2 agonists, indicating that microglial cells are involved in the altered expression of *NPY* after SE infection. Surely this does not exclude the potential contribution of other neuronal cells to NPY level in the CNS. NPY works in an autocrine fashion via its receptor, NPY 1R, to control the microglia neuroinflammatory response (35). This is consistent with our observation that exogenous NPY reduced the TNF-α related inflammatory response in TLR2-challenged microglial cells, similar to its previously documented effects on LPS-induced microglial production of IL-1β (38). Enteral feeding with colostrum dampened the neuroinflammation (reduced leukocyte count and CRP levels), but did not affect the NPY level in the CSF, suggesting that dietary modulation of neuroinflammation did not affect cerebral NPY production. Nevertheless, our data suggest that exogenous NPY may have the potential to suppress neuroinflammation in preterm neonates.

IL-18 is a pro-inflammatory cytokine, and the levels of it in CSF and plasma increase during bacterial meningitis (39) and sepsis (40). In our study, the CSF and plasma levels of IL-18 increased in SE piglets relative to Controls, as did the physiological inhibitor, IL-18 BP (34), in the CSF. The *IL-18* transcription level increased only in PVWM and the hippocampus of SE piglets, not in the prefrontal cortex. In isolated preterm pig microglial cells, *IL-18* transcription increased following TLR2 stimulation. Together, these confirm the cerebral contribution to the elevated IL-18 level in the CSF during SE infection. The elevated IL-18 BP level in CSF, probably recruited from the systemic circulation, may exacerbate the intracranial inflammation, as IL-18 BP can neutralise the action of IL-18 in counteracting invading bacteria. Enteral feeding reduced the infection-induced IL-18 response, as shown by lowered abundance of IL-18 and IL-18 BP in CSF and plasma and decreased *IL-18* transcription in brain tissues.

Neuroinflammation involving IL-18 affects membrane-type matrix metalloproteinases (MT-MMPs) (18). CSF abundance of MMP-14, a MT-MMP, and its transcription in the brain tissues increased in SE-infected piglets relative to the Controls, in accordance with other studies (41). Being a proteinase-cleaving extracellular matrix (ECM) protein, MMP-14 inreases its level to digest the tight junction of the blood–CSF barrier and basement membrane to allow immune cells to enter the brain parenchyma to attack invading bacteria (42); increased levels of leukocytes and platelets were also observed in this study. Our results suggested that this MMP-14 regulation in the CNS involved the activation of microglia cells via TLR2. Furthermore, no clear effect of the enteral feeding on MMP-14 abundance in the CSF or its transcription in brain tissues was observed. Together with the limited effects of enteral feeding of colostrum on other proteins in the CSF, this suggests that early enteral feeding mainly has an impact on systemic inflammation but not on neuroinflammation.

Among the early response proteins in plasma, eight showed differential abundance as early as 6 h post-infection, and these included haptoglobin, Serpin A3-6, Serpin A3-8, vWF, LBP, sCD14, MBL1, and APOA4 (Table S4). Haptoglobin, LBP, and the serpins in particular responded with a clear linear increase in plasma levels over the first 24 h after SE infection, suggesting their high relevance as early markers of sepsis and bacterial infection. On the other hand, enteral feeding increased, rather than reduced, the levels of these proteins (plus sCD14 and ApoA4) in plasma. Thus, the immune-dampening effect of enteral feeding is not mediated by a reduction in haptoglobin, LBP, serpins, sCD14, and ApoA4 in plasma, and colostrum may even stimulate their synthesis, independently of any direct effect on inflammation. By contrast, enteral feeding reduced the level of vWF, a coagulation factor, as early as 6 h after infection. The level of vWF was negatively correlated with mean platelet counts, confirming that vWF localises platelets to the endothelial surface in sepsis (43). Plasma vWF is reported to be more active in infants than in adults, and vWF can be the first haematological sign of sepsis (44). Collectively, the results suggest that the plasma vWF level may serve as an early indicator of sepsis as well as the immunomodulatory effects of early colostrum feeding.

Among the plasma proteins related to development, metabolism, and protein processing, 14 proteins were affected at 12 h, with 13 affected by SE and only three by enteral feeding with colostrum. At 24 h, 33 of 46 proteins were affected by SE infection and 21 by enteral feeding. None of the 46 CSF proteins, except IL-18, were affected by enteral feeding. Combined with the observation of immune response proteins, this supports that enteral feeding for 24 h had limited short term effects on protein levels in the CSF after SE infection. During sepsis, multiple complement proteins may enter the CNS and engage in neuroinflammation (45). It was not until 12 h after SE infection that plasma complement factors (e.g., C1QA, C1R, and C5) changed in concentration and other complement factors (C2, C4BPA, C5, and C8B) were affected at 24 h. The small activation fragments affected by SE infection, such as C3, C4a, and C5a, increase the permeability of blood vessels and attract leukocytes (8, 20, 21). The complement system also plays a role in eliminating invading bacteria and regulating the immune response to CNS infection by another Gram-positive bacteria, *Streptococcus pneumonia* (46). C1q, MBL1, and properdin may play a role in initiating the lectin and alternative complement pathways. Considering that MBL1 showed a difference already at 6 h, while C1q and properdin showed differences at 12 and 24 h, it is possible that there is a time-dependent activation of the three complement pathways in sepsis.

Many of the proteins that responded to SE infection and enteral feeding with colostrum warrant further investigation, including NPY, IL-18, and MMP-14, due to their roles in neuroinflammation, microglia activation, and blood–CSF barrier disruption. It is possible that exogenous NPY may be used to dampen neuroinflammation in newborns. Two of the detected proteins in plasma, IL-18 and sCD14, show a differential response to Gram-positive and Gram-negative sepsis, with IL-18 levels being highest in Gram-positive sepsis (47) and sCD14 highest in Gram-negative sepsis (34). Combining these two proteins could render us a tool for early differentiation between Gram-positive and Gram-negative sepsis. Compared with classical blood biomarkers of systemic inflammation (e.g., CRP), the proteins detected in this study, including haptoglobin, vWF, MBL1, MRC1, sCD14, and LBP, showed a very early response to infection, indicating their potential in serving as new early markers of infection or sepsis.

The preterm pig is the only model of sepsis in preterm neonates that incorporates all the normal clinical signs of prematurity, such as impaired immunity, respiratory distress, and metabolic dysfunctions. It is highly relevant to study piglet response to bacterial infection during the immediate postnatal adaptation phase, which represents a highly sensitive period for infections and maladaptation in preterm infants. However, it cannot be excluded that the brain-related response to SE and enteral feeding with bovind colsotrum would have been more pronounced after longer exposure (beyond the first 24 h) to SE and enteral feeding. Due to the extremely high technical demand of this pig model, a non-inoculated but colostrum-fed group was skipped. It remains to be investigated whether the effect of colostrum feeding observed in this study is specific to the highly bioactive bovine colostrum or if it is a general effect of enteral feeding, independent of the diet type. Cerebral and systemic responses may also vary according to gestational age at birth, postnatal age, type of pathogen, and infection intensity. Clearly, both pre-clinical and clinical studies are required to clarify the several variables that affect the pathological response to systemic bacterial infection and sepsis in preterm neonates. Combined with proteomic analyses of plasma and CSF, our animal model has shown a new path to investigate both mechanisms and clinically relevant markers of sepsis in preterm neonates.

## Conclusion

Our findings in this study may reflect how systemic infection by Gram-positive bacteria soon after birth may affect brain development and neuroinflammation in preterm infants. *S. epidermidis* infection induced rapid changes in inflammation-related CSF and the plasma proteome, and early enteral feeding dampened the SE-induced changes in the plasma proteome, but with limited effect in the CSF. The affected plasma proteins may serve as new early biomarkers of Gram-positive systemic infection in newborns, which may also affect central aspects of neuroinflammation, such as microglia activation and blood–CSF barrier disruption.

## Data Availability Statement

The mass spectrometry proteomics data have been deposited to the ProteomeXchange Consortium via the PRIDE partner repository with the dataset identifier PXD016013.

## Ethics Statement

All experimental animal protocols were performed in accordance with the Danish Animal Welfare Act and this study was approved by the Danish Committee for Animal Research.

## Author Contributions

TM conceived the study, analyzed the proteomic data, performed qPCR and ELISA experiments, and prepared the manuscript. AS performed the proteomics analysis and protein annotation. SP performed the primary cell culture. AB and DN performed the animal experiment, sample collection, and assisted in interpreting the data. PS conceived the study, interpreted the data, and prepared the manuscript. P-PJ conceived the study, analyzed and interpreted the data, and prepared the manuscript. All authors drafted the work, revised it critically for important intellectual content, and approved the final version.

### Conflict of Interest

The authors declare that the research was conducted in the absence of any commercial or financial relationships that could be construed as a potential conflict of interest.

## Supplementary Material

Figure S1 Microglia purity and NPY 1R response.

Table S1 Clinical data of piglets included in the proteomics analysis.

Table S2 Number of proteins with differential abundance in CSF and plasma.

Table S3 Proteins with differential abundance in CSF.

Table S4 Proteins with differential abundance in plasma.

Table S5 Primer sequence of selected genes.

Data Sheets S1 and S2 Proteomics information for identification.

## Abbreviations

APOA4: apolipoprotein A-IV
APPs: antimicrobial proteins and peptides
aSOFA: SOFA score adapted to preterm piglets
BCB: blood-CSF barrier
C1QA: complement C1q A chain
C1R: complement C1r
C5: complement C5
CFU: colony-forming units
CNS: central nervous system
CoNS: coagulase-negative staphylococci
CRP: C-reactive protein
CSF: cerebrospinal fluid
ECM: extracellular matrix
FDR: false discovery rate
GA: gestational age
HP: haptoglobin
HPX: hemopexin
IgG: immunoglobulin G
IHC: immunohistochemistry
IL-18 BP: IL-18 binding protein
IL-18: interleukin 18
LBP: LPS-binding protein
LOS: late-onset sepsis
LPS: lipopolysaccharide
LTA-SA: lipoteichoic acid from *S. aureus*
MBL1: mannose-binding lectin (protein A) 1
MMP-14: matrix metalloproteinases 14
MS: mass spectrometry
MT-MMPs: membrane-type matrix metalloproteinases
NPY 1R: neuropeptide Y receptor type 1
NPY: neuropeptide Y
Pref cortex: prefrontal cortex
PVWM: paraventricular white matter
SAA: serum amyloid A
sCD14: soluble CD14
SE: *Staphylococcus epidermidis*
SEM: standard error of the mean
SOFA: sequential organ failure assessment
TLR: toll-like receptor
TLR2: toll-like receptor 2
TLR4: toll-like receptor 4
TNF-α: tumour necrosis factor-α
VCAM1: vascular cell adhesion protein 1
vWF: von Willebrand factor.
