# Supplementary material for: Rapid Proteome Changes in Plasma and Cerebrospinal Fluid Following Bacterial Infection in Preterm Newborn Pigs
Source: Front Immunol. 2019 Nov 15;10:2651. doi: 10.3389/fimmu.2019.02651 (PMC6873289; doi:10.3389/fimmu.2019.02651)
Supplement: Figure S1 — Microglia purity and NPY 1R response. [file Data_Sheet_3.pdf]

Supplemental information

Rapid proteome changes in plasma and cerebrospinal fluid following bacterial infection in preterm newborn pigs

Tik Muk, Allan Stensballe, Stanislava Pankratova, Duc Ninh Nguyen, Anders Brunse, Per Torp Sangild, Ping-Ping Jiang

Figure S1. Microglia purity and *NPY 1R* response

Table S1. Clinical data of piglets included in the proteomics analysis

Table S2. Number of proteins with differential abundance in CSF and plasma

Table S3. Proteins with differential abundance in CSF

Table S4. Proteins with differential abundance in plasma

Table S5. Primer sequence of selected genes

Figure S1 Microglia purity and NPY 1R response

A

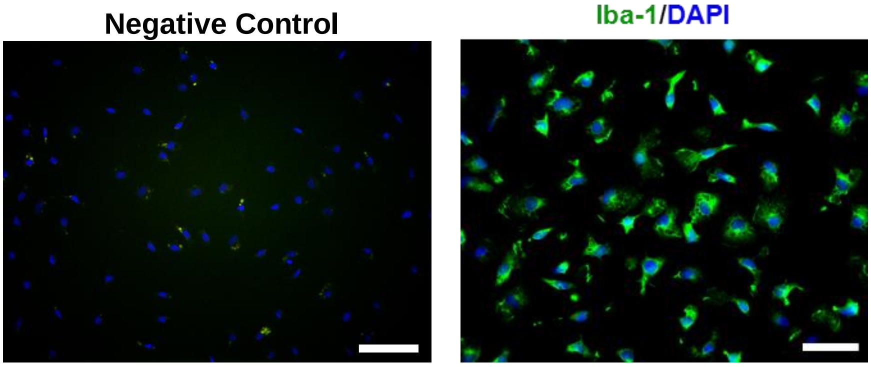

B

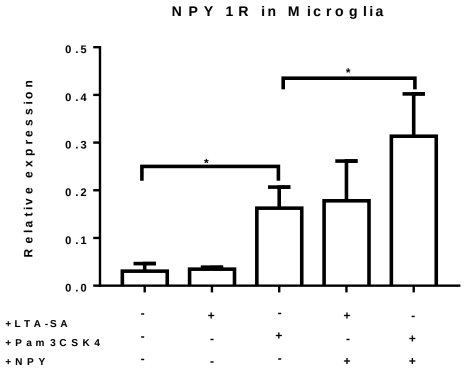

Figure S1A. Immunofluorescent staining of purified porcine microglial culture. Mixed glial culture was prepared from porcine hippocampi and grown for 11-14 days *in vitro*. Microglia cells were then isolated by shaking the mixed culture at 250 RPM, plated on poly-L-lysine-coated slide and grown for 24 h. Cells were stained with anti-Iba1 antibodies (ionized calcium binding adaptor molecule 1; green) and total nuclei were counterstained with DAPI (blue). The microglia culture was 90% pure, as determined by ratio of Iba-1–positive cells to total nuclei. Scale bar, 20 µm. **Figure S1B.** In the primary cultures of porcine microglial cells, stimulation with TLR2 agonists (LTA-SA or Pam3CSK4) for 24 h increased transcription levels of *NPY 1R* (only for Pam3CSK, $p < 0.05$). Treatment with exogenous NPY (0.5 µM) up-regulated the transcription levels of *NPY 1R* (vs. Pam3CSK4 alone, $p < 0.05$).

Table S1. Clinical data of the piglets included in the proteomics analysis

	Treatment Groups		
	Control	SE	SE+ENT
aSOFA score (range)	2 (1 - 2)[a]	4 (3 - 5)[b]	3 (1 - 3)[a,b]
CSF total leukocytes (cells/µL)	4.9 ± 1.2[a]	44.2 ± 3.9[b]	24.3 ± 4.9[c]
CSF Bacteriology	0 ± 0[a]	1.86 ± 0.62[b]	0.28 ± 0.28[a]
CSF-plasma fluorescein ratio (%)	2.9 (2.6 - 3.8)[a]	6.1 (3.7 - 8.7)[b]	4.2 (2.8 - 4.6)[a,b]
CRP in blood (mg/L)	0.016 ± 0.003[a]	0.7 ± 0.2[b]	0.9 ± 0.2[b]
Blood fibrinogen (mg/L)	1.9 ± 0.1[a]	3.9 ± 0.4[b]	4.4 ± 0.2[b]
Blood bacteriology (*SE* CFU/ml)			
6 h	0 ± 0[a]	2.26 ± 0.58[b]	2.13 ± 0.69[a,b]
12 h	0 ± 0[a]	2.67 ± 0.61[b]	2.20 ± 0.81[a,b]
24 h	0 ± 0[a]	2.24 ± 0.52[b]	0.96 ± 0.52[a,b]
Blood total leukocytes ($\times 10^{9}$ cells/L)			
6 h	3.1 ± 0.1[a]	1.4 ± 0.2[b]	2.2 ± 0.2[c]
12 h	2.9 ± 0.2[a]	1.5 ± 0.3[b]	1.8 ± 0.2[b]
24 h	2.3 ± 0.2[a]	1.6 ± 0.2[b]	1.7 ± 0.2[a]
Blood platelets ($\times 10^{9}$ cells/L)			
6 h	219.2 ± 2.3[a]	210.5 ± 2.2[b]	218.3 ± 3.1[a]
12 h	221.6 ± 2.5[a]	213.8 ± 2.8[b]	225.5 ± 3.0[a]
24 h	230.2 ± 2.0[a,b]	217.8 ± 5.4[b]	232.6 ± 2.7[a]

aSOFA, adapted Sepsis-related Organ Failure Assessment, presented as median (quantile); CRP, C-reactive protein; *In vivo* blood-CSF barrier permeability measured by CSF-serum fluorescein ratio. Data presented as mean ± SEM or median (1st - 3rd quantile); data bearing same letter are not significantly different from each other.

Table S2. Number of proteins with differential abundance ($q \leq 0.10$) in CSF and plasma

	SE vs. Control	SE+ENT vs. Control	SE+ENT vs. SE
**CSF**	83	27	0
**24 h plasma**	80	16	65
**12 h plasma**	38	3	19
**6 h plasma**	8	0	5

Table S3. Proteins with differential abundance in CSF

			Abundance[1]			*q* value		
**UniProt ID**	**Gene**	**Protein Name**	**Control (n = 8)**	**SE (n = 9)**	**SE+ENT (n = 10)**	**SE/ Control**	**SE+ENT/ Control**	**SE+ENT/ SE**
		**Neurodevelopment Related**						
F1ST92	NPY	Neuropeptide Y	22.85 ± 0.21	22.24 ± 0.29	22.59 ± 0.27	0.100	0.896	0.997
F1S9C6	MMP14	Matrix metalloproteinase 14	15.82 ± 2.74	20.76 ± 2.33	18.64 ± 0.22	0.075	0.107	0.174
F1RFS7	MMP15	Matrix metalloproteinase 15	16.35 ± 0.37	18.90 ± 0.33	19.26 ± 0.29	0.002	0.043	0.997
F1RWV2	TIMP1	Metalloproteinase inhibitor 1	21.97 ± 0.13	26.00 ± 0.09	25.90 ± 0.13	0.015	0.379	0.997
F1SAE9	LAMB1	Laminin subunit β 1	24.93 ± 0.16	24.68 ± 0.24	24.72 ± 0.25	0.098	0.285	0.997
I3L812	TPP1	Tripeptidyl peptidase 1	21.64 ± 0.15	18.16 ± 0.18	16.46 ± 0.16	0.089	0.301	0.997
F1SK62	ROBO1	Roundabout guidance receptor 1	19.23 ± 0.35	21.2 ± 0.31	19.17 ± 0.3	0.078	0.221	0.997
F1SB50	RELN	Reelin	27.13 ± 0.27	26.24 ± 0.2	26.21 ± 0.27	0.003	0.002	0.997
ADML	ADM	ADM	21.30 ± 0.1	22.54 ± 0.11	22.23 ± 0.16	0.008	0.110	0.997
D0G6X8	HEXB	β-hexosaminidase	23.45 ± 0.33	22.21 ± 0.23	22.41 ± 0.12	0.000	0.003	0.997
I3L8N9	GRIA4	Glutamate ionotropic receptor AMPA type subunit 4	21.02 ± 0.1	19.76 ± 0.17	20.2 ± 0.19	0.033	0.368	0.997
F1SBR6	OLFML3	Olfactomedin 3	26.25 ± 0.19	25.55 ± 0.15	25.71 ± 0.09	0.000	0.000	0.960
I3LA84	PLOD3	Procollagen-lysine,2-oxoglutarate 5-dioxygenase 3	23.39 ± 0.14	22.41 ± 0.23	22.87 ± 0.17	0.003	0.340	0.997
F1SPG5	LOC100738 595	Fibulin 2	25.05 ± 0.11	24.21 ± 0.09	24.26 ± 0.11	0.002	0.003	0.997
F1S889	PDYN	Proenkephalin-B (ob in uniprot)	23.4 ± 2.54	22.72 ± 0.18	23.14 ± 1.97	0.000	0.516	0.327
7B2	SCG5	C-terminal peptide	22.71 ± 2.54	21.91 ± 2.17	22.74 ± 2.05	0.055	0.896	0.261
F1S431	AARS	cytoplasmic Alanine--tRNA ligase	20.27 ± 0.19	19.63 ± 2.23	22.66 ± 1.98	0.060	0.896	0.997
F1RU22	EFEMP2	EGF containing fibulin-like extracellular matrix protein 2	25.58 ± 0.1	24.98 ± 0.17	25.17 ± 0.12	0.033	0.327	0.997
F1SJL8	IQCJ-SCHIP1	IQCJ-SCHIP1, Schwannomin interacting protein 1	21.9 ± 0.18	24.1 ± 0.14	19.27 ± 0.2	0.073	0.211	0.997
I3L571	LOC100738	Follistatin-like 5	21.47 ± 3.21	20.63 ± 0.33	16.79 ± 0.26	0.066	0.826	0.997

	432							
F1SHP1	ADAMTS1	ADAM metallopeptidase with thrombospondin type 1 motif 1	24.34 ± 0.08	25.06 ± 0.16	24.92 ± 0.12	0.003	0.036	0.997
D7RK08	TFRC	Transferrin receptor protein 1	19.48 ± 0.16	18.23 ± 2.43	20.18 ± 0.16	0.005	0.135	0.997
F1RUM1	AFM	Afamin	26.28 ± 0.18	27.03 ± 0.23	26.40 ± 0.2	0.033	0.896	0.606
		**Acute-phase Response**						
A5PF01	BF	Properdin	30.17 ± 3.25	30.92 ± 2.17	30.95 ± 2.65	0.003	0.002	0.997
F1SLV6	C1R	Complement C1r	26.26 ± 0.46	27.77 ± 0.37	27.68 ± 0.44	0.000	0.001	0.997
F1SBS4	C3	Complement C3	28.87 ± 2.7	29.85 ± 0.33	29.42 ± 0.23	0.004	0.317	0.997
F1RQW2	C4A	Complement Component 4a	30.76 ± 0.09	31.33 ± 0.11	31.22 ± 0.14	0.098	0.321	0.997
F1SME1	C5	Complement Component 5a	24.59 ± 0.18	25.56 ± 0.15	25.19 ± 0.18	0.015	0.336	0.997
I3LF89	CPN2	Carboxypeptidase N subunit 2	15.69 ± 0.09	22.01 ± 0.09	21.79 ± 0.12	0.087	0.377	0.997
F1SJW8	SERPING1	Plasma protease C1 inhibitor precursor	29.31 ± 0.13	29.93 ± 0.11	29.56 ± 0.24	0.055	0.896	0.997
HPT	HP	Haptoglobin	17.39 ± 0.29	23.94 ± 0.33	27.93 ± 0.15	0.000	0.000	0.997
ITIH1	ITIH1	Inter-α-trypsin inhibitor heavy chain H1	26.46 ± 0.25	27.25 ± 0.22	26.7 ± 0.26	0.054	0.896	0.976
F1RUM4	ITIH2	Inter-α-trypsin inhibitor heavy chain H2	28.2 ± 0.15	29.06 ± 0.25	28.47 ± 0.1	0.045	0.896	0.960
F1SH92	ITIH4	Inter-α-trypsin inhibitor heavy chain H4	29.09 ± 0.36	29.99 ± 0.22	29.81 ± 0.18	0.003	0.049	0.997
ITIH4	ITIH4	Inter-α-trypsin inhibitor heavy chain H4	28.3 ± 3.57	29.22 ± 2.88	29.05 ± 0.44	0.008	0.058	0.997
F1SCC6	LOC100153 899	serpin A3-8	22.53 ± 0.72	26.33 ± 0.62	27.15 ± 0.53	0.000	0.000	0.997
F1SCC7	LOC396684	A Serpin family member	23.17 ± 0.29	25.9 ± 0.31	26.74 ± 0.23	0.000	0.000	0.997
F1S9B9	SAA2	Serum amyloid A 2	ND	21.12 ± 0.1	21.37 ± 2.05	0.000	0.000	0.997
FCN1	FCN1	Fibrinoge ficolin-1	ND	19.26 ± 0.31	18.58 ± 3.06	0.000	0.000	0.997
F1SLX2	A2M	α-2-macroglobulin	25.78 ± 0.1	26.97 ± 0.21	26.64 ± 0.18	0.005	0.221	0.997
F1RJE3	VWA1	Von Willebrand factor A domain containing 1	19.69 ± 0.13	19.11 ± 0.21	15.41 ± 0.19	0.068	0.420	0.997
F1SL22	VWF	von Willebrand factor	23.48 ± 0.14	25.33 ± 0.17	25.12 ± 0.18	0.000	0.000	0.997
		**Immune Response**						
A2SW51	CD14	CD14	26.75 ± 0.13	27.18 ± 0.16	27.35 ± 0.17	0.089	0.005	0.997
I3LA67	IL-18BP	Interleukin-18 binding protein	ND	22.01 ± 0.19	21.30 ± 0.11	0.000	0.000	0.005
F1S571	COL11A1	Collagen type 11 α-1-chain	21.98 ± 0.07	18.86 ± 0.06	18.79 ± 0.06	0.050	0.001	0.997
F1SSE7	COL15A1	Collagen type 15 α-1-chain	23.35 ± 0.22	22.75 ± 0.13	22.83 ± 0.13	0.054	0.151	0.997
I3LBZ1	COL18A1	Collagen type 18 α-1-chain	25.85 ± 0.11	25.41 ± 0.33	25.32 ± 0.21	0.050	0.101	0.997

I3L6Q6	F13B	Coagulation factor 13 B chain	22.23 ± 0.53	20.56 ± 0.51	22.65 ± 0.32	0.003	0.678	0.908
I3L5U6	LBP	LPS binding protein	21.69 ± 0.27	22.73 ± 0.34	23.58 ± 0.19	0.069	0.000	0.908
F1SIB1	F2	Prothrombin	27.41 ± 0.49	28.15 ± 0.36	27.61 ± 0.27	0.064	0.896	0.908
F1S715	FUCA2	α-L-fucosidase 2	26.83 ± 0.17	26.42 ± 0.25	26.32 ± 0.15	0.100	0.022	0.997
F1RMN7	HPX	Hemopexin	23.05 ± 0.32	25.13 ± 0.44	25.16 ± 0.26	0.055	0.151	0.997
F1SFI5	HRG	Histidine rich glycoprotein	29.64 ± 0.14	30.29 ± 0.26	29.87 ± 2.66	0.078	0.896	0.908
F1S567	VCAM1	Vascular cell adhesion protein 1 precursor	23.88 ± 0.1	24.69 ± 0.13	24.61 ± 0.16	0.000	0.001	0.997
F1SCV9	LOC100519 252 (APOB)	Apolipoprotein B-100	27.15 ± 0.1	28.17 ± 0.12	27.78 ± 0.12	0.015	0.336	0.997
F1RJI1	MLEC	Malectin	18.85 ± 0.28	20.93 ± 0.14	21.18 ± 2	0.033	0.497	0.997
F1SFI4	KNG1	Kininogen 1	26.87 ± 2.52	27.44 ± 0.15	26.92 ± 0.08	0.095	0.896	0.876
F1RN44	LAMP1	Lysosomal associated membrane glycoprotein 1	21.63 ± 3.23	20.69 ± 0.14	20.27 ± 1.97	0.000	0.000	0.997
F1STI0	CNTN5	Contactin 5	18.98 ± 3.34	18.26 ± 2.23	19.34 ± 2.03	0.083	0.896	0.908
I3LQA4	PROCR	Endothelial protein C receptor	20.35 ± 2.35	20.93 ± 0.25	20.63 ± 1.98	0.087	0.896	0.997
F1S6B5	FMOD	Fibromodulin	25.93 ± 0.07	24.36 ± 0.09	24.89 ± 0.11	0.032	0.616	0.997
F1RFN9	FSCN1	Fascin	24.9 ± 0.22	24.39 ± 0.14	24.61 ± 0.11	0.010	0.659	0.997
F1S0A2	LOC100737 887	peptidylprolyl isomerase B, PPIB	23.64 ± 0.09	22.08 ± 0.1	23.1 ± 0.1	0.054	0.896	0.982
F1RWA3	MRC1	Macrophage mannose receptor 1	17.55 ± 0.11	21.23 ± 0.11	18.69 ± 0.07	0.020	0.433	0.997
		**Metabolism**						
F1SI88	HEXA	β-hexosaminidase	22.38 ± 0.2	21.54 ± 2.69	21.57 ± 1.84	0.055	0.194	0.997
F1S8N1	HGFAC	Hepatocyte growth factor activator	23.05 ± 0.15	24.18 ± 0.19	23.99 ± 0.14	0.056	0.187	0.997
F1SEH4	HTRA1	HtrA serine peptidase 1	22.01 ± 0.13	21.58 ± 0.17	19.44 ± 0.12	0.066	0.216	0.997
F1RKC7	PLBD2	Phospholipase B	20.37 ± 2.62	15.06 ± 0.11	17.44 ± 0.17	0.001	0.002	0.997
I3LNS9	FUCA1	α-L-fucosidase precursor	22.04 ± 0.14	21.51 ± 0.19	21.18 ± 0.18	0.033	0.001	0.997
F1RLR8	GGT5	γ-glutamyltransferase 5	22.73 ± 0.18	21.81 ± 0.18	22.06 ± 0.1	0.004	0.152	0.997
F1RP34	APOA1BP	NAD(P)H-hydrate epimerase	21.65 ± 2.58	20.79 ± 0.22	21.36 ± 2.03	0.089	0.896	0.976
		**Protein Processing**						
F1RRV7	ST3GAL1	CMP-N-acetylneuraminate-β-galactosamide-α-2,3-sialyltransferase 1	20.35 ± 2.78	17.45 ± 0.31	19.60 ± 0.2	0.078	0.112	0.997
I3LGM7	PTPRR	Protein tyrosine phosphatase receptor type R	19.54 ± 0.22	21.61 ± 0.19	22.04 ± 0.13	0.089	0.896	0.997

A7U5U2	RPS27A	60S ribosomal protein L40	20.69 ± 0.11	23.26 ± 0.09	23.44 ± 0.18	0.064	0.896	0.976
		**Others**						
ACYP1	ACYP1	Acylphosphatase-1	21.48 ± 0.1	20.58 ± 0.22	21.22 ± 0.19	0.089	0.896	0.908
I3LUC8	LOC100154783	Peptidyl-prolyl cis-trans isomerase B	23.76 ± 0.27	22.52 ± 2.06	23.32 ± 0.17	0.045	0.896	0.976
I3LM06	LOC102167652	Follistatin like 5	21.81 ± 2.97	20.94 ± 0.44	18.98 ± 0.49	0.033	0.142	0.997
F1RXR1	SRPX	Sushi repeat containing protein, X-linked	22.04 ± 0.21	20.86 ± 0.13	20.85 ± 0.15	0.001	0.001	0.997
I3L999	RARRES2	Retinoic acid receptor responder 2	25.03 ± 0.33	24.24 ± 2.16	24.27 ± 2.62	0.028	0.068	0.997
F1RYS0	FRAS1	Fraser extracellular matrix complex subunit 1	20.14 ± 0.16	21.79 ± 0.07	22.35 ± 0.1	0.069	0.758	0.997
F1SUA0	FBLN7	Fibulin 7	15.89 ± 0.17	18.34 ± 0.11	16.74 ± 2.44	0.073	0.652	0.997
F1S6M4	CDH15	Cadherin 15	18.97 ± 0.32	20.92 ± 0.15	18.64 ± 0.15	0.034	0.003	0.997
F1SFF3	NID2	Nidogen 2	26.89 ± 0.21	26.24 ± 0.29	26.41 ± 0.27	0.000	0.006	0.997
I3LPA5	TMEM132C	Transmembrane protein 132C	24.93 ± 2.74	24.24 ± 2.33	24.26 ± 0.22	0.075	0.101	0.997
ALBU	ALB	Serum albumin	31.28 ± 0.37	31.91 ± 0.33	31.6 ± 0.29	0.098	0.896	0.976

[1]**,** Abundance is base-2 logarithm transformed. 2, ND, not detected.

Table S4. Proteins with differential abundance in plasma

			**Abundance[1] 6 h**			***q* value 6 h**			**Abundance[1] 12 h**			***q* value 12 h**			**Abundance[1] 24 h**			***q* value 24 h**		
**UniProt ID**	**Gene**	**Protein Name**	**Control**	**SE**	**SE+ENT**	**SE/Control**	**SE+ENT/Control**	**SE+ENT/SE**	**Control**	**SE**	**SE+ENT**	**SE/Control**	**SE+ENT/Control**	**SE+ENT/SE**	**Control**	**SE**	**SE+ENT**	**SE/Control**	**SE+ENT/Control**	**SE+ENT/SE**
		**Acute-phase Response**																		
F1SCC6	LOC100153899	serpin A3-8	24.72 ± 0.97	29.08 ± 0.35	29.62 ± 0.33	0	0.997	0	27.07 ± 0.80	31.47 ± 0.40	31.87 ± 0.26	0	0.98	0	27.77 ± 0.36	32.11 ± 0.40	33.26 ± 0.13	0	0.098	0
K7GQ21	MBL1	Mannose-binding protein A	25.26 ± 0.28	24.29 ± 2.38	24.00 ± 3.01	0.06	0.997	0.297												
VWF	VWF	von Willebrand factor	27.11 ± 0.14	28.02 ± 0.13	27.78 ± 0.11	0.069	0.997	0	26.89 ± 0.12	27.66 ± 0.15	27.08 ± 0.12	0.804	0.233	0.015	22.47 ± 2.63	24.00 ± 0.29	22.87 ± 2.68	0.633	0.212	0.009
F1SCC7	LOC100156325	serpin A3-6	28.07 ± 0.16	29.04 ± 0.17	29.29 ± 0.15	0	0.997	0.008	28.74 ± 0.22	30.31 ± 0.19	30.44 ± 0.20	0	0.98	0	28.78 ± 0.12	31.00 ± 0.26	31.80 ± 0.19	0	0.075	0
HPT	HP	Haptoglobin	28.12 ± 0.27	29.24 ± 0.17	29.57 ± 0.14	0	0.997	0.008	29.24 ± 0.26	30.83 ± 0.17	31.47 ± 0.30	0	0.98	0.003	28.57 ± 0.21	31.16 ± 0.27	32.51 ± 0.18	0	0.008	0
F1SMI8	C6	Complement 6							21.03 ± 0.23	21.40 ± 0.15	22.03 ± 1.53	0.019	0.824	0.925	21.20 ± 0.18	22.10 ± 1.84	22.78 ± 2.66	0.002	0.582	0.127
C1QA	C1QA	Complement C1q A-chain							22.45 ± 0.24	21.88 ± 2.07	23.32 ± 1.62	0.124	0.007	0.824	22.79 ± 0.31	22.92 ± 1.39	24.21 ± 2.10	0	0.008	0.737
I3L6K3	CRP	C-reaction protein							23.81 ± 0.46	25.04 ± 2.35	25.28 ± 2.79	0.021	0.98	0.156	24.30 ± 2.12	25.43 ± 2.50	25.94 ± 2.27	0.002	0.901	0.026
F1SET0	FGL1	Fibrinogen-like 1							24.13 ± 0.12	24.45 ± 0.23	24.90 ± 0.16	0.084	0.98	0.925	24.03 ± 0.12	25.01 ± 0.19	25.38 ± 0.13	0	0.637	0.001
F1S567	VCAM1	Vascular cell adhesion molecule-1							24.87 ± 0.09	25.38 ± 0.10	25.05 ± 0.06	0.556	0.276	0.003	24.57 ± 0.13	25.52 ± 0.10	24.94 ± 0.18	0.18	0.056	0
F1SLV6	C1R	Complement C1r							25.38 ± 0.18	26.37 ± 0.18	26.46 ± 0.28	0.019	0.98	0.093	25.40 ± 0.18	27.60 ± 0.25	28.02 ± 0.15	0	0.648	0
F1RWW8	F13A1	Coagulation factor XIII A chain							26.76 ± 0.10	26.48 ± 0.10	26.32 ± 0.13	0.07	0.98	0.691						

F1RMN7	HPX	Hemopexin	26.86 ± 0.21	27.52 ± 0.13	27.91 ± 0.26	0.015	0.98	0.468	27.09 ± 0.18	27.90 ± 0.28	28.53 ± 0.13	0	0.402	0.075
CBG	Serpina6	Corticosteroid-binding globulin	28.45 ± 0.14	28.14 ± 0.12	27.96 ± 0.09	0.01	0.98	0.371	28.15 ± 0.12	27.72 ± 0.10	27.49 ± 0.11	0.001	0.693	0.051
F1SM61	FBLN1	Fibulin-1	29.45 ± 0.08	29.03 ± 0.09	28.96 ± 0.10	0.003	0.98	0.03	29.05 ± 0.07	28.90 ± 0.06	28.61 ± 0.12	0.006	0.222	0.605
CO3	C3	Complement 3	31.54 ± 0.07	31.94 ± 0.09	31.87 ± 0.09	0.112	0.98	0.051						
TTHY	TTR	Transthyretin	32.51 ± 0.06	32.33 ± 0.07	32.19 ± 0.09	0.004	0.98	0.342	32.15 ± 0.07	31.90 ± 0.07	31.75 ± 0.07	0.003	0.685	0.104
F1SN68	ORM1	A-1-acid glycoprotein	35.85 ± 0.05	35.75 ± 0.06	35.60 ± 0.04	0.019	0.98	0.911						
F1RQW7	C2	Complement 2							20.37 ± 0.27	23.28 ± 0.42	23.96 ± 0.52	0	0.851	0
SAMP	APCS	Serum amyloid P-component							24.26 ± 0.39	24.93 ± 0.57	27.17 ± 0.21	0.001	0.056	0.721
F1S0J2	C4BPA	C4-binding protein α							24.80 ± 0.11	25.51 ± 0.13	25.31 ± 0.15	0.06	0.901	0.004
F1S790	C8B	Complement C8-β chain							26.21 ± 0.13	25.72 ± 0.12	26.09 ± 0.10	0.666	0.31	0.038
F1RZN7	KLKB1	Kallikrein-B1							27.16 ± 0.07	26.71 ± 0.08	26.71 ± 0.07	0.002	0.901	0.001
F1SC80	RBP4	Retinol-binding protein 4							27.97 ± 0.09	27.28 ± 0.12	26.99 ± 0.16	0	0.626	0.004
F1SME1	C5	Complement 5							28.12 ± 0.08	28.62 ± 0.11	28.83 ± 0.08	0	0.391	0.003
I3LJA6	FGA	Fibrinogen-α-chain							28.20 ± 0.38	28.94 ± 0.23	29.77 ± 0.13	0	0.188	0.295
F1SCE3	SERPINA5	Serpin family A member 5							29.22 ± 0.07	28.77 ± 0.10	28.86 ± 0.07	0.06	0.901	0.009
ITIH1	ITIH1	Inter-α-trypsin inhibitor heavy chain H1							29.40 ± 0.10	29.26 ± 0.11	28.98 ± 0.08	0.044	0.389	0.737

F1RXM6	SERPINA7	Thyroxine-binding globulin													29.78 ± 0.08	29.57 ± 0.07	29.36 ± 0.12	0.016	0.582	0.399
F1RKY2	SERPIND1	Serpin family D member 1													29.95 ± 0.10	29.55 ± 0.10	29.54 ± 0.11	0.07	0.901	0.064
F1SJW8	SERPING1	Serpin family G member 1													30.38 ± 0.10	30.76 ± 0.10	30.71 ± 0.13	0.116	0.901	0.099
Q9GMA6	SERPINA3-2	Serpin family A member 3													30.44 ± 0.08	30.86 ± 0.12	31.12 ± 0.05	0	0.432	0.016
K7GQ48	A2M	α-2-macroglobulin													30.87 ± 0.11	30.71 ± 0.09	30.50 ± 0.12	0.076	0.829	0.498
F1SH92	ITIH4	Inter-α-trypsin inhibitor heavy chain 1													31.47 ± 0.11	32.08 ± 0.13	32.24 ± 0.15	0	0.88	0
A5PF01	BF	Properdin													31.80 ± 0.05	32.20 ± 0.07	32.13 ± 0.08	0.005	0.901	0
I3LN42	GC	Vitamin D-binding protein													32.02 ± 0.08	31.90 ± 0.08	31.69 ± 0.08	0.059	0.539	0.695
ITIH4	ITIH4	Inter-α-trypsin inhibitor heavy chain H4													32.31 ± 0.08	32.93 ± 0.10	33.09 ± 0.12	0.002	0.901	0.013
F1RX36	LOC100514666	Fibrinogen-α-chain													32.36 ± 0.31	33.15 ± 0.16	33.79 ± 0.10	0	0.284	0.047
F1RX35	LOC100627396	Fibrinogen-γ-chain-like													32.53 ± 0.30	33.47 ± 0.14	34.00 ± 0.09	0	0.352	0.004
F1RX37	FGB	Fibrinogen-β-chain													33.15 ± 0.32	34.07 ± 0.16	34.59 ± 0.11	0	0.424	0.019
B3CL06	TF	Serotransferrin													34.15 ± 0.07	33.89 ± 0.06	33.74 ± 0.06	0.001	0.634	0.04
F1S9B9	SAA2	Serum amyloid A2													ND	26.19 ± NA	25.94 ± NA	0	0	0.021
		**Development**																		
F1SPG5	LOC100738595	fibulin-2							23.23 ± 2.70	22.52 ± 2.11	22.37 ± 2.46	0.002	0.98	0.023						

F1S3G6	OLFM2	Olfactomedin-2 (brain)							23.46 ± 0.16	22.74 ± 0.18	22.48 ± 2.11	0.01	0.98	0.161	23.33 ± 0.12	23.04 ± 1.40	22.61 ± 1.97	0.008	0.368	0.475
I3LHF9	OLFM1	Olfactomedin-1 (brain)							24.46 ± 0.20	24.10 ± 1.68	23.62 ± 0.13	0.015	0.971	0.88						
F1S7K2	LRG1	Leucine rich α-2-glycoprotein 1							25.49 ± 0.14	25.89 ± 0.10	26.05 ± 0.13	0.041	0.98	0.465	26.03 ± 0.09	26.36 ± 0.18	26.81 ± 0.09	0.006	0.349	0.452
F1SHP1	ADAMTS1	ADAM metallopeptidase with thrombospondin type 1 motif 1													22.88 ± 0.17	23.88 ± 0.19	23.25 ± 2.02	0.42	0.223	0.002
Q1KS52	ALS	Alsin Rho guanine nucleotide exchange factor													23.05 ± 0.14	22.42 ± 1.36	22.94 ± 1.99	0.67	0.098	0.004
F1SUE2	OMD	Osteomodulin													23.11 ± 0.14	22.46 ± 0.09	22.95 ± 1.99	0.628	0.122	0.002
Q9XSH0	FOLR1	Folate receptor 1													23.23 ± 2.05	24.51 ± 2.42	24.94 ± 2.19	0.036	0.901	0.17
F1SI93	NEO1	Neogenin 1													23.33 ± 0.10	23.07 ± 0.16	22.71 ± 1.97	0.041	0.554	0.597
VINC	VCL	Vinculin													23.34 ± 2.03	22.87 ± 0.17	22.23 ± 1.94	0.011	0.389	0.465
F1SFM5	CNTN3	Contactin 3													23.51 ± 0.09	23.42 ± 1.42	23.02 ± 2.00	0.076	0.402	0.737
F1RF11	MMP2	Matrix metallopeptidase 2													24.26 ± 0.14	23.50 ± 0.16	23.96 ± 0.17	0.427	0.249	0.003
F1SHN1	CNTN1	Contactin 1													24.28 ± 0.07	23.98 ± 0.10	23.81 ± 0.18	0.065	0.901	0.321
F1SFM2	CHL1	Cell adhesion molecule L1-like													24.56 ± 0.19	23.93 ± 1.46	23.30 ± 2.71	0	0.212	0.058
F1SB67	IGF2R	Insulin-like growth factor 2 receptor													24.79 ± 0.13	24.38 ± 0.14	24.21 ± 0.08	0.012	0.737	0.216

K7GRK7	TNXB	Tenascin XB													25.06 ± 0.11	24.88 ± 0.09	24.64 ± 0.12	0.06	0.634	0.611
F1SDX9	PRDX2	Peroxiredoxin-2													25.60 ± 0.18	25.86 ± 0.20	25.03 ± 0.17	0.19	0.098	0.726
F1SGY4	NELL2	Neural EGFL like 2													25.86 ± 0.07	25.59 ± 0.07	25.35 ± 0.13	0.002	0.37	0.247
F1SM72	NCAM1	Neural cell adhesion molecule 1													26.02 ± 0.12	25.96 ± 0.09	25.65 ± 0.07	0.044	0.135	0.737
I3LD86	PGLYRP2	N-acetylmuramoyl-L-alanine amidase (brain)													27.75 ± 0.08	27.47 ± 0.07	27.54 ± 0.06	0.216	0.901	0.072
F1SRC8	CLEC3B	C-type lectin domain family 3 member B													29.34 ± 0.10	28.96 ± 0.09	29.02 ± 0.08	0.124	0.901	0.046
		**Immune Response**																		
F1RWA3	MRC1	Mannose receptor C-type 1	22.19 ± 0.19	23.43 ± 1.93	22.72 ± 2.45	0.53	0.553	0							22.16 ± 1.93	23.24 ± 0.18	22.78 ± 1.98	0.095	0.432	0
I3L5U6	LBP	LPS-binding protein	24.61 ± 0.15	25.09 ± 0.11	25.35 ± 0.13	0.007	0.997	0.324	25.12 ± 0.07	25.82 ± 0.13	26.22 ± 0.15	0	0.824	0.019	25.16 ± 0.15	26.11 ± 0.14	26.65 ± 0.11	0	0.135	0
A2SW51	CD14	CD14	27.82 ± 0.09	28.05 ± 0.08	28.24 ± 0.08	0.062	0.997	0.896							28.11 ± 0.04	27.99 ± 0.08	28.34 ± 0.05	0.111	0.028	0.61
F1S285	COL14A1	Collagen type XIV α-1-chain							23.48 ± 0.19	22.74 ± 1.57	22.76 ± 0.17	0.021	0.98	0.052						
F1SIE1	LOC100621838	immunoglobulin superfamily containing leucine-rich repeat protein-like							23.68 ± 0.18	23.20 ± 0.21	22.91 ± 1.59	0.094	0.98	0.736						
F1RYI8	COL3A1	Collagen type III α-1-chain							24.80 ± 0.15	23.74 ± 0.23	23.76 ± 0.20	0.01	0.98	0.019	25.24 ± 0.15	23.99 ± 0.28	24.18 ± 2.11	0.033	0.901	0.004
I3LNM9	LOC100624077	endogenous retrovirus group V member 2 Env							24.97 ± 0.15	23.74 ± 1.65	24.18 ± 0.17	0.005	0.98	0	24.94 ± 0.10	24.21 ± 1.48	24.77 ± 2.88	0.666	0.297	0.023

		polyprotein												
I3LSV6	COL2A1	Collagen type II α-1-chain	24.98 ± 0.23	23.87 ± 1.66	24.30 ± 1.69	0.252	0.98	0.036						
F1RW32	SPARCL1	SPARC-like 1	25.33 ± 0.27	24.79 ± 0.21	24.26 ± 0.24	0.007	0.98	0.548	24.39 ± 0.33	24.22 ± 0.23	23.32 ± 2.03	0.06	0.284	0.737
BGH3	TGFBI	TGF-β-induced protein ig-h3	26.07 ± 0.19	24.99 ± 0.31	25.02 ± 0.18	0.049	0.98	0.093	26.12 ± 0.13	25.05 ± 1.41	24.66 ± 0.19	0	0.726	0.003
F1RS37	POSTN	Periostin	26.16 ± 3.05	25.00 ± 1.74	24.82 ± 1.72	0.01	0.98	0.066						
F1S021	COL5A1	Collagen type V α-1-chain	26.79 ± 0.08	26.55 ± 0.13	26.12 ± 0.14	0.011	0.779	0.925	26.18 ± 0.20	26.16 ± 0.18	25.48 ± 0.18	0.094	0.222	0.737
I3LUR7	COL6A3	Collagen type VI α-3-chain	28.24 ± 0.09	27.64 ± 0.12	27.71 ± 0.12	0.02	0.98	0.019	28.34 ± 0.09	27.80 ± 0.07	27.63 ± 0.14	0	0.755	0.002
F1RQH9	CD109	CD109	28.53 ± 0.07	28.35 ± 0.07	28.28 ± 0.06	0.055	0.98	0.496	28.35 ± 0.10	28.29 ± 0.07	28.00 ± 0.08	0.044	0.233	0.737
TGBR3	TGFBR3	TGF-β receptor type 3							23.49 ± 0.14	23.13 ± 1.92	22.86 ± 1.99	0.063	0.864	0.452
F1RHA7	TGFBI	TGF-β-induced protein ig-h3							23.61 ± 0.15	22.85 ± 0.18	23.31 ± 0.27	0.608	0.648	0.096
I3LHW8	LOC100622782	Collectin subfamily member 10							23.62 ± 0.15	23.16 ± 0.12	23.14 ± 2.01	0.079	0.901	0.075
Q4Z8N7	PAF-AH	Platelet-activating factor-acetylhydroxylase							24.20 ± 0.17	23.48 ± 1.43	24.23 ± 2.11	0.671	0.129	0.047
F1SMF4	ITGA2	Integrin subunit α2							24.31 ± 0.13	23.76 ± 0.08	23.45 ± 2.04	0	0.609	0.03
D0G7G0	SPP1	Osteopontin							25.03 ± 0.32	26.16 ± 0.20	25.68 ± 0.14	0.211	0.693	0.009
F1SEY1	LOC100518647	Mannosidase-α-class 2B member 1							26.50 ± 0.07	26.43 ± 0.07	25.85 ± 0.24	0.015	0.105	0.737
I3LQM5	PROS1	Protein S (α)							26.96 ± 0.10	26.58 ± 0.10	26.45 ± 0.11	0.011	0.901	0.083
I3L	PROC	Vitamin K-							28.15 ±	27.69 ±	27.73 ±	0.116	0.901	0.063

RJ4		dependent protein C													0.10	0.12	0.14			
F1SK70	LOC100625463	Protein S													28.30 ± 0.05	28.04 ± 0.08	28.13 ± 0.07	0.336	0.901	0.088
GELS	GSN	Gelsolin													29.24 ± 0.15	28.52 ± 0.19	28.64 ± 0.20	0.166	0.901	0.069
F1SFI4	KNG1	Kininogen 1													29.68 ± 0.06	29.56 ± 0.08	29.06 ± 0.17	0.002	0.064	0.737
F1RII7	HBB	Haemoglobin subunit β													30.79 ± 0.18	31.03 ± 0.20	30.20 ± 0.14	0.141	0.064	0.726
F1SCV9	LOC100519252	Apolipoprotein B-100-like													32.22 ± 0.09	31.75 ± 0.14	31.67 ± 0.13	0.03	0.901	0.046
F1SFI5	HRG	Histidine rich glycoprotein													32.36 ± 0.07	31.94 ± 0.11	32.19 ± 0.09	0.42	0.609	0.026
		**Metabolism**																		
APOA4	APOA4	Apolipoprotein A-IV	28.42 ± 0.14	28.30 ± 0.13	29.43 ± 0.16	0.001	0	0.983	28.48 ± 0.16	28.33 ± 0.12	29.88 ± 0.20	0	0	0.925	28.82 ± 0.12	28.39 ± 0.15	29.95 ± 0.15	0	0	0.24
F1SKB1	CP	Ceruloplasmin							20.94 ± 1.90	25.64 ± 0.43	25.93 ± 0.55	0	0.98	0	21.74 ± 1.91	27.72 ± 0.56	28.44 ± 0.34	0	0.648	0
F1RQY6	SLC4A1	solute carrier family 4 member-1							21.37 ± 0.24	21.97 ± 0.34	22.82 ± 1.59	0.019	0.82	0.925						
F1SC70	CTSA	Carboxypeptidase							22.60 ± 2.63	22.81 ± 2.14	23.07 ± 2.16	0.02	0.98	0.89	22.8 ± 2.65	22.76 ± 1.88	23.22 ± 2.02	0.05	0.056	0.737
I3LK59	ENO1	Enolase							24.23 ± 0.31	23.51 ± 0.25	23.12 ± 1.60	0.038	0.98	0.554						
F1S1G8	AOC3	Amine oxidase							25.38 ± 0.19	24.96 ± 0.17	24.58 ± 0.17	0.006	0.98	0.468						
I3LQH7	BLVRB	Biliverdin reductase B													23.61 ± 0.27	24.31 ± 0.32	23.01 ± 2.00	0.383	0.056	0.321
F1RYU5	ART3	NAD(P)(+)-arginine ADP-ribosyltransferase													24.43 ± 0.14	23.73 ± 0.12	23.66 ± 0.10	0	0.901	0
F1SAS3	LOC100157	Adipocyte plasma													24.92 ± 0.09	24.69 ± 0.09	24.38 ± 2.12	0.019	0.539	0.456

	249	membrane-associated protein (found on monocytes)																		
I3LGB2	PCSK9	Proprotein convertase subtilisin type 9													25.09 ± 0.19	24.41 ± 0.16	23.98 ± 0.35	0.013	0.864	0.151
CATL1	CTSL	Cathepsin L1													25.23 ± 0.13	24.40 ± 0.22	24.84 ± 2.16	0.485	0.695	0.048
F1RUE4	GPLD1	Glycosylphosphatidylinositol specific phospholipase D1													25.40 ± 0.18	25.39 ± 0.16	24.71 ± 0.16	0.06	0.135	0.737
I3LF89	CPN2	Carboxypeptidase N subunit 2													26.13 ± 0.09	26.71 ± 0.13	26.99 ± 0.06	0	0.553	0.005
F1S568	EXTL2	Exostosin-like glycosyltransferase 2													26.13 ± 0.11	25.99 ± 0.07	25.81 ± 0.10	0.095	0.619	0.735
F1SQX9	APOD	Apolipoprotein D													29.10 ± 0.11	28.47 ± 0.12	28.87 ± 0.10	0.363	0.233	0.002
F1RK01	CPB2	Carboxypeptidase B2													29.18 ± 0.09	28.90 ± 0.05	28.96 ± 0.07	0.179	0.901	0.046
APOC3	APOC3	Apolipoprotein C-III													30.37 ± 0.13	29.60 ± 0.15	30.03 ± 0.20	0.416	0.493	0.014
APOE	APOE	Apolipoprotein E													32.06 ± 0.07	31.39 ± 0.13	31.33 ± 0.15	0.002	0.901	0.004
A0A0C3	APOA1	Apolipoprotein A-I													34.65 ± 0.07	34.34 ± 0.08	34.24 ± 0.10	0.008	0.901	0.048
		**Other**																		
ACTS	ACTA1	Actin, α-skeletal muscle							24.25 ± 0.19	23.89 ± 0.18	23.45 ± 2.58	0.054	0.98	0.832						
I3LG75	LOC102162699	MLV-related proviral Env polyprotein-like							25.15 ± 0.08	24.63 ± 0.11	24.64 ± 0.12	0.02	0.98	0.039	24.92 ± 0.09	24.26 ± 0.13	24.17 ± 0.18	0.005	0.901	0.012
F1S	CILP2	Cartilage							25.56 ±	25.04 ±	24.93 ±	0.012	0.98	0.122	25.07 ±	24.46 ±	24.61 ±	0.19	0.901	0.038

6Q3		intermediate layer protein-2							0.13	0.10	0.14				0.09	0.17	2.86			
F1SEY8	PIGR	Polymeric immunoglobulin receptor													ND	ND	23.10 ± NA	0.997	0	0
I3LCF9	AHSP	α-hemoglobin stabilizing protein													21.46 ± 2.50	22.12 ± 2.17	20.78 ± 2.42	0.276	0.044	0.375
F1SD87	FBLN5	Fibulin-5													23.81 ± 0.13	23.30 ± 1.42	23.62 ± 0.07	0.54	0.582	0.046
F1S902	COMP	Cartilage oligomeric matrix protein													24.61 ± 0.17	24.26 ± 0.11	23.74 ± 0.20	0.002	0.3	0.308
I3LEE6	PCOLCE	Procollagen C-endopeptidase enhancer													26.33 ± 0.10	26.21 ± 0.09	25.82 ± 0.12	0.014	0.209	0.737
CADH5	CDH5	Cadherin-5													28.56 ± 0.13	28.47 ± 0.08	28.09 ± 0.11	0.027	0.219	0.737
		**Protein Processing**																		
I3LFP1	LOC100739735	chymotrypsinogen B-like	21.96 ± 0.28	22.53 ± 1.38	23.24 ± 1.87	0.062	0.997	0.983	22.34 ± 1.95	22.95 ± 1.60	23.99 ± 1.66	0	0.035	0.496	22.01 ± 2.56	23.19 ± 2.27	23.67 ± 2.76	0.002	0.851	0.039
D0G7F6	TPI1	Triosephosphate isomerase													23.19 ± 0.33	22.91 ± 2.27	21.95 ± 1.91	0.087	0.389	0.737
I3LGL1	PRIM1	DNA primase													24.09 ± 2.09	23.03 ± 2.50	23.19 ± 2.02	0.046	0.901	0.012
F1S3Q8	VNN3	Vanin 3													24.71 ± 0.25	24.71 ± 0.17	23.67 ± 0.19	0.007	0.028	0.737
F1SGH0	PTPRG	Protein tyrosine phosphatase, receptor type G													25.18 ± 0.09	24.95 ± 0.09	24.77 ± 0.10	0.019	0.693	0.321

[1], Abundance is base-2 logarithm transformed. 2, ND, not detected.

Table S5. Primer sequence of selected genes

Symbol	Name	Primer Sequences	
		Forward	Reverse
HPRT1	Hypoxanthine-guanine phosphoribosyltransferase (REF)	ACACTGGCAAAACAATGCAA	TGCAACCTTGACCATCTTTG
IL-18	Interleukin-18	AACCAGGGACATCAAGCCGT	CTGCACAGAGATGGTTACTGC
MMP14	Matrix metalloproteinase-14	TCACATCGGGTTACCCTTCC	CCACCTTGGGGGTGTAGTTC
NPY	Neuropeptide Y	ACTACTCGGCGTTGAGACAT	ACCACACAGAAGGGTCTTCG
NPY 1R	Neuropeptide Y receptor Y1	TGTCTACACGCTGATGGACC	CTATTACTCGGCCTCCAGCC
